# Clinical and Molecular Spectrum of Myotonia and Periodic Paralyses Associated With Mutations in *SCN4A* in a Large Cohort of Italian Patients

**DOI:** 10.3389/fneur.2020.00646

**Published:** 2020-07-29

**Authors:** Lorenzo Maggi, Raffaella Brugnoni, Eleonora Canioni, Paola Tonin, Veronica Saletti, Patrizia Sola, Stefano Cotti Piccinelli, Lara Colleoni, Paola Ferrigno, Antonella Pini, Riccardo Masson, Fiore Manganelli, Daniele Lietti, Liliana Vercelli, Giulia Ricci, Claudio Bruno, Giorgio Tasca, Antonio Pizzuti, Alessandro Padovani, Carlo Fusco, Elena Pegoraro, Lucia Ruggiero, Sabrina Ravaglia, Gabriele Siciliano, Lucia Morandi, Raffaele Dubbioso, Tiziana Mongini, Massimiliano Filosto, Irene Tramacere, Renato Mantegazza, Pia Bernasconi

**Affiliations:** ^1^Neuroimmunology and Neuromuscular Diseases, Fondazione IRCCS Istituto Neurologico Carlo Besta, Milan, Italy; ^2^Section of Clinical Neurology, Department of Neurosciences, Biomedicine and Movement Sciences, University of Verona, Verona, Italy; ^3^Developmental Neurology Unit, Fondazione IRCCS Istituto Neurologico Carlo Besta, Milan, Italy; ^4^Clinica Neurologica, Azienda Ospedaliero Universitaria di Modena, Modena, Italy; ^5^Unit of Neurology, Center for Neuromuscular Diseases, ASST Spedali Civili and University of Brescia, Brescia, Italy; ^6^SC Neurologia e Stroke Unit, Azienda Ospedaliera Brotzu, Cagliari, Italy; ^7^Child Neurology and Psychiatry Unit, IRCCS Istituto delle Scienze Neurologiche di Bologna, Bologna, Italy; ^8^Department of Neurosciences, Reproductive Sciences and Odontostomatology, University of Naples “Federico II”, Naples, Italy; ^9^Pediatric Unit, Ospedale Valduce, Como, Italy; ^10^Department of Neurosciences Rita Levi Montalcini, University of Turin, Turin, Italy; ^11^Department of Clinical and Experimental Medicine, University of Pisa, Pisa, Italy; ^12^Center of Translational and Experimental Myology, Istituto Giannina Gaslini, Genova, Italy; ^13^Unità Operativa Complessa di Neurologia, Dipartimento di Scienze Dell'Invecchiamento, Neurologiche, Ortopediche e della Testa-Collo, Fondazione Policlinico Universitario A. Gemelli IRCCS, Rome, Italy; ^14^Fondazione IRCCS Casa Sollievo della Sofferenza, Laboratory of Medical Genetics, San Giovanni Rotondo, Italy; ^15^Department of Experimental Medicine, Sapienza University of Rome, Rome, Italy; ^16^Dipartimento Materno-Infantile, S.C. Neuropsichiatria Infantile, Presidio Ospedaliero Provinciale Santa Maria Nuova, IRCCS di Reggio Emilia, Reggio Emilia, Italy; ^17^Department of Neurosciences, University of Padova, Padova, Italy; ^18^Emergency Neurology, IRCCS Mondino Foundation, Pavia, Italy; ^19^Research and Clinical Development Department, Scientific Directorate, Fondazione IRCCS Istituto Neurologico Carlo Besta, Milan, Italy

**Keywords:** myotonia, periodic paralysis, SNEL, channelopathies, voltage-gated sodium channel Na_V_1.4, *SCN4A* gene mutation

## Abstract

**Background:** Four main clinical phenotypes have been traditionally described in patients mutated in SCN4A, including sodium-channel myotonia (SCM), paramyotonia congenita (PMC), Hypokaliemic type II (HypoPP2), and Hyperkaliemic/Normokaliemic periodic paralysis (HyperPP/NormoPP); in addition, rare phenotypes associated with mutations in SCN4A are congenital myasthenic syndrome and congenital myopathy. However, only scarce data have been reported in literature on large patient cohorts including phenotypes characterized by myotonia and episodes of paralysis.

**Methods:** We retrospectively investigated clinical and molecular features of 80 patients fulfilling the following criteria: (1) clinical and neurophysiological diagnosis of myotonia, or clinical diagnosis of PP, and (2) presence of a pathogenic SCN4A gene variant. Patients presenting at birth with episodic laryngospasm or congenital myopathy-like phenotype with later onset of myotonia were considered as neonatal SCN4A.

**Results:** PMC was observed in 36 (45%) patients, SCM in 30 (37.5%), Hyper/NormoPP in 7 (8.7%), HypoPP2 in 3 (3.7%), and neonatal SCN4A in 4 (5%). The median age at onset was significantly earlier in PMC than in SCM (*p* < 0.01) and in Hyper/NormoPP than in HypoPP2 (*p* = 0.02). Cold-induced myotonia was more frequently observed in PMC (*n* = 34) than in SCM (*n* = 23) (*p* = 0.04). No significant difference was found in age at onset of episodes of paralysis among PMC and PP or in frequency of permanent weakness between PP (*n* = 4), SCM (*n* = 5), and PMC (*n* = 10). PP was more frequently associated with mutations in the S4 region of the NaV1.4 channel protein compared to SCM and PMC (*p* < 0.01); mutations causing PMC were concentrated in the C-terminal region of the protein, while SCM-associated mutations were detected in all the protein domains.

**Conclusions:** Our data suggest that skeletal muscle channelopathies associated with mutations in SCN4A represent a continuum in the clinical spectrum.

## Introduction

The *SCN4A* gene on chromosome 17q23 encodes the α-subunit of the voltage-gated sodium channel Na_V_1.4, responsible for the generation of action potentials and excitation of skeletal muscle fibers. The sodium channel is constituted by α subunits associated with β subunits (1). The α subunit is a single polypeptide chain that folds to four homologous but non-identical repeats (repeats I to IV). Each repeat contains six transmembrane segments (S1–S6). When inserted in the membrane, the four repeats form a central pore with segments five and six lining its wall, while the segment S4 is the channel voltage sensor due to positively charged amino acids. The loop (*P* loop) between S5 and S6 forming the extracellular domain is responsible for ion selectivity (1). The activation of the channel generates an action potential (AP) and the fast inactivation after AP can prevent repetitive discharge, which assures the physiological excitability changes of sarcolemma and the normal skeletal muscle contraction. Mutations in *SCN4A* lead to changes in skeletal muscle excitability, which is connected with the activation or inactivation speed of muscle ion channels (1). These mutations are responsible for a wide spectrum of clinical manifestations, ranging from myotonia to periodic paralysis (PP) and recently discovered phenotypes such as severe neonatal episodic laryngospasms, severe fetal hypokinesia or classical congenital myopathy, myalgia, and exercise intolerance, congenital myasthenic syndrome, and sudden infant death syndrome (2–4). However, four main clinical phenotypes have been traditionally described in patients mutated in *SCN4A* based on myotonia and paralysis features, including sodium-channel myotonia (SCM) and paramyotonia congenita (PMC) considered as non-dystrophic myotonias (NDM) and characterized by increased skeletal muscle excitability, and Hypokalemic type II (HypoPP2) and Hyperkalemic/Normokalemic PP (HyperPP/NormoPP), instead associated with reduced excitability. All three phenotypes represent a continuum in the clinical spectrum and their combined prevalence has been estimated in 0.5 per 100,000 in UK (5). However, only scarce data have been reported in literature on large cohorts of patients including all the aforementioned phenotypes, most of the studies being focused on single clinical subgroups (6, 7). Here, we describe the clinical, neurophysiological, and molecular features of a large cohort of Italian patients affected by skeletal muscle channelopathies associated with *SCN4A* mutations.

## Materials and Methods

### Patients

In this retrospective study, 80 patients genetically diagnosed in our laboratory at Fondazione IRCCS Istituto Neurologico Carlo Besta since 2004 were included fulfilling the following criteria: (1) clinical and neurophysiological diagnosis of myotonia, or clinical diagnosis of PP, and (2) presence of a pathogenic *SCN4A* gene variant. We also included two familial asymptomatic cases mutated in *SCN4A*. Clinical phenotypes were defined on the basis of predominant symptom (myotonia vs. periodic paralysis); in the myotonia subgroup, we identified PMC or SCM according to the presence of paradoxical myotonia or warm-up phenomenon, respectively. PP were classified as HyperPP/NormoPP and Hypo PP2 according to the potassium levels during paralytic attacks. Furthermore, patients presenting at birth with severe neonatal episodic laryngospasm (SNEL) or congenital myopathy-like phenotype and later onset of myotonia were considered as neonatal *SCN4A*. Pattern of muscle weakness was defined according to neurological examination at the end of the follow-up period.

The local ethics committees approved the study. All patients, parents/guardians provided written informed consent for genetic analysis and use of their anonymized clinical data at the time of their first visit at the individual centers.

### Genetic Analyses

Direct Sanger sequencing of the *SCN4A* 24 exons was performed for 62 patients on genomic DNA extracted from peripheral blood as previously reported (8).

For 20 patients a targeted next generation sequencing panel covering the *SCN4A* gene was used. The panel was designed by Sure Design (https://earray.chem.agilent.com/suredesign/) (Agilent Technologies). DNA libraries were prepared using the HaloPlex Target Enrichment System (Agilent Technologies), following the manufacturer's instructions, and sequenced on the MiSeq Illumina (Illumina, San Diego, CA, USA). Alignment to human genome assembly hg19 (GRCh37) was carried out and Binary alignment/map (BAM file) and variant call format (VCF file) was generated. The variants were annotated with the free web server wANNOVAR (http://wannovar.usc.edu/) to generate the VCF files. To identify pathogenic variants and to exclude variants with allele frequency more than 1% public database (i.e., dbSNP, 1000 Genome project, ExAC, ClinVar, and HGMD) were used. To predict the functional effect of a novel variant Mutation Taster (http://www.mutationtaster.org/), PolyPhen-2 (http://genetics.bwh.harvard.edu/pph2/), Proven (http://provean.jcvi.org/genome_submit_2.php), and Human Splicing Finder (http://www.umd.be/HSF3/) were consulted.

All the NGS-discovered pathogenic variants were validated by Sanger sequencing on an ABI3500Dx DNA Analyzer (Thermo Fisher Scientific) in the DNA of the patient and, if any, the parents.

### Statistics

Continuous variables were expressed as means with standard deviations and medians with value ranges, while categorical variables were expressed as numbers and percentages. Associations between variables were assessed by the Mann-Whitney test or Fisher exact test, as appropriate. *P* < 0.05 were considered statistically significant and all tests were two-sided. STATA statistical software, version 15 (StataCorp. 2017. Stata Statistical Software: Release 15. College Station, TX: StataCorp LLC) was used for the statistical analysis.

## Results

We observed a PMC phenotype in 36/80 (45%) patients, SCM in 30 (37.5%), PP in 10 (12.5%), and neonatal *SCN4A* in 4 (5%), with a male/female ratio of 1.36. The median age at onset was 6 years (range 0–48) and the median disease duration was 22.5 years (range 2–61). Clinical features according to phenotypes are shown in Table 1. In addition, two familial asymptomatic patients were included, carrying the p.F1298C and p.A1156S mutations, respectively; the two mutations were associated with SCM in the probands. Despite not having symptoms or signs related to myotonia, both the subjects, aged 65 and 89 years at the end of the follow-up, showed myotonic discharges on electromyography. Fifty-six out of 82 (68.3%) patients were familial cases belonging to 20 different pedigrees. Phenotype concordance was observed in all families, except for the two aforementioned families including an asymptomatic subject, one family with the proband showing a neonatal SCN4A, and the mother displaying a mild SCM and two siblings carrying the p.M1592V mutation affected by Hyper/NormoPP and SCM, respectively. Electromyography response to short and long exercise test and cooling of muscle (9, 10) was performed in 26/80 (32.5%) patients, resulting in agreement with clinical phenotype in almost all the patients; in particular 15 PMC showed a pattern I, 8 SCM a pattern III, and 2 Hyper/NormoPP a pattern IV. The only exception was a patient displaying a neonatal *SCN4A and* showing a pattern III, suggestive of SCM, but with clear clinical paradoxical myotonia; a similar discrepancy has already been reported (11).

**Table 1:** Clinical features according to phenotype.

	**Whole cohort**	**NDM**	**PMC**	**SCM**	**PP**	**Hyper/**	**HypoPP2**	**Neonatal**	***p*-value**
						**NormoPP**			
N° (%)	80	66 (82.5)	36 (45)	30 (37.5)	10 (12.5)	7 (8.7)	3 (3.7)	4 (5)	
M/F	1.36/1	1.36/1	1.25/1	1.5/1	2.33/1	1.33/1	3/0	1/1	0.96
Median age at onset (y, range)	6 (0–48)	8 (0.3–48)	5.2 (0.3–48)	14.2 (1.5–48)	7.75 (1–33)	5.5 (1–10)	15.5 (15–33)	Birth	**<0.01^a^**
Cold-induced myotonia (%)	58 (72.5)	57 (86.4)	34 (94.4)	23 (76.7)	1 (10)	1 (14.3)	0	3 (75)	
Painful myotonia (%)	18 (22.5)	14 (21.2)	4 (11.1)	10 (33.3)	0	0	0	0	
Lower limb myotonia (%)	39 (48.7)	37 (56.1)	16 (44.4)	21 (70)	1 (10)	1 (14.3)	0	1 (25)	0.07
Handgrip myotonia (%)	53 (66.2)	49 (74.2)	28 (77.8)	21 (70)	3 (30)	3 (42.9)	0	1 (25)	0.13
Cranial myotonia (%)	56 (70)	51 (77.3)	30 (83.3)	21 (70)	2 (20)	2 (28.6)	0	3 (75)	0.17
Episodes of paralyses (%)	33 (41.2)	23 (34.8)	23 (63.9)	0	10 (100)	7 (100)	3 (100)	0	
Permament weakness (%)	21 (26.2)	15 (22.7)	10 (27.8)	5 (16.7)	4 (40)	4 (57.1)	0	2 (50)	0.24
Muscle hypertrophy (%)	37 (46.2)	29 (43.9)	13 (36.1)	17 (56.7)	5 (50)	5 (71.4)	0	3 (75)	0.20
Mexiletine benefit (n° treated pts)^b^	27 (43)	24 (40)^c^	10 (19)	14 (21)	0	0	0	3 (3)	
Acetazolamide benefit (n° treated pts)^b^	11 (20)	6 (12)	6 (12)	0	5 (8)	3 (6)	2 (2)	0	

a *Neonatal cases were excluded from this analysis*.

b *Data on treatment were available only for 53 patients*.

c *Mexiletine was stopped in 1st days of treatment in four patients due to side effects*.

*NDM, non-dystrophic myotonias; PMC, paramyotonia congenita; SCM, sodium channel myotonia; PP, periodic paralysis; HyperPP/NormoPP, hyperkaliemic/normokaliemic periodic paralysis; HypoPP2, hypokaliemic periodic paralysis type II; M, male; F, female; y, years; pts, patients*.

*Bold values resulted significant*.

### Non-dystrophic Myotonias

PMC (*n* = 36) was slightly more frequent than SCM (*n* = 30). Median age at onset was significantly (*p* < 0.01) earlier in PMC (5.2 years; range 0.3–48) than in SCM (14.2 years; range 1.5–48). Myotonia in NDM involved more frequently cranial muscles (*n* = 51), followed by hand (*n* = 49), and lower limb muscles (*n* = 37), especially at thigh level. Lower limb muscles were more frequently involved in SCM (*n* = 21) than in PMC (*n* = 16) (*p* = 0.05); although not significant, cranial muscle (*p* = 0.09) and handgrip myotonia (*p* = 0.59) were slightly more frequent in PMC than in SCM (see Table 1 for details). Cold-induced myotonia was more frequently observed in PMC (*n* = 34) than in SCM (*n* = 23) (*p* = 0.04). Painful myotonia tended to be more frequent in SCM (*n* = 10) than in PMC (*n* = 4), although not statistically significant. Permanent weakness was detected in 10/36 (27.8%) PMC and 5/30 (16.7%) SCM (*p* = 0.41), mainly in cranial (*n* = 8), thigh (*n* = 8), neck flexor (*n* = 6), and distal upper limb (*n* = 5) muscles. Of note, distal upper limb weakness was detected only in PMC. Muscle weakness was mild to moderate in most of the cases (Medical Research Council muscle power scale: MRC 3-4/5) in both NMD and PP. Muscle hypertrophy was evident in 13 (36.1%) PMC and 17 (56.7%) SCM (*p* = 0.15), mainly at calves (*n* = 18), and thigh (*n* = 12), while generalized hypertrophy with Hercules-like appearance was observed in 5 (16.7%) SCM and 3 (8.3%) PMC. Twenty-three out of 36 (63.9%) PMC patients had PP presenting at a median age of 5.5 years (range 1–20) and a median time from myotonia to PP onset of 0 years (range 0–15), without any case with PP preceding myotonia presentation. Of note, 1 SCM carrying the p.A699T mutation had stridor presenting in adult age. Triggering factors for episodes of paralysis in PMC were mainly cold temperature (*n* = 17), prolonged exercise (*n* = 14), and rest after exercise (*n* = 9). Data on treatment were available for 53 (66.2%) NDM patients; mexiletine was administered in 21/30 (70%) SCM and 19/36 (52.8%) PMC patients with benefit in 14 (66.7%) and 10 (71.4%), respectively; four out of 40 NDM patients stopped mexiletine due to side effects in 1st days of treatment. Acetazolamide was given to 12/36 (33.3%) PMC patients, with benefit in 6 (50%). Further seven patients had benefit from lamotrigine (*n* = 3), carbamazepine (*n* = 2), buprenorphine (*n* = 1), and propafenone (*n* = 1); all of them had PMC, except for the three patients taking lamotrigine, who displayed a SCM phenotype.

### Periodic Paralyses

The median age at onset of PP was 7.75 years (range 1–33), not significantly different from age at onset of episodes of paralysis in PMC (*p* = 0.72). Hyper/NormoPP was observed in 7 (70%) patients and HypoPP2 in 3 (30%). Median age at onset was significantly (*p* = 0.02) earlier in Hyper/NormoPP (5.5 years; range 1–10) than in HypoPP2 (15.5 years; range 15–33). Five (50%) patients, all with Hyper/NormoPP, also had myotonia, with warm-up phenomenon, worsened by cold in only one case and never reported as painful. No significant difference was found in muscle distribution and age at onset of myotonia in PP and in NDM. Median time from PP and myotonia onset was 5 years (range 0–21); of note, PP onset always preceded myotonia presentation. Triggering factors for Hyper/Normo were mainly post-exercise period (*n* = 5) and cold temperature (*n* = 4), for HypoPP2 post-exercise period (*n* = 2). Episodes of paralysis were usually generalized in 3/7 (42.9%) Hyper/NormoPP and in all HypoPP2 or limited to lower limbs in 4/7 (57.1%) Hyper/NormoPP. Duration of PP was usually more than 24 h in all HypoPP2 patients and in 2/7 (28.6%) Hyper/NormoPP and <24 h in remaining 5 (71.4%) Hyper/NormoPP, in most of the cases lasting 1–4 h.

Permanent weakness was found in 4/10 (40%) patients, all affected by Hyper/NormoPP, and its occurrence was not statistically different from that in NDM; weakness was limited to thigh muscles in all patients, except for one with also involvement of distal upper limb muscles. Muscle hypertrophy was found in 5/7 (71.4%) Hyper/NormoPP patients and its frequency was not significantly different among PP and NDM.

Data on treatment were available for 8/10 (80%) PP patients; acetazolamide was administered in 6/7 (85.7%) Hyper/NormoPP and 2/3 (66.7%) HypoPP2, with benefit in 3 (50%) and 2 (100%), respectively.

### Neonatal SCN4A

Three out of 4 (75%) patients with neonatal phenotype had typical SNEL and one had congenital myopathy-like presentation with bilateral clubfoot, hip dislocation, facial dysmorphism in association with myotonia characterized by warm-up phenomenon, and absence of stridor, previously reported in literature (12). This patient had an affected mother, carrying the same mutation p.N1180I and showing a SCM, with onset of hand and facial myotonia at the age of 12 years; mother's neurological examination revealed mild handgrip myotonia, diffuse muscle hypertrophy, pes cavus, hyporeflexia, and nasal speech. Notably, two SNEL patients were mother and son, respectively, carrying the p.I693T mutation and presenting with laryngeal stridor and hypotonia at birth, followed by paradoxical myotonia of facial and bulbar muscles at the age of 6 months followed by muscle hypertrophy. Stridor was triggered by cold temperature or cold food or beverages and spontaneously disappeared in adolescence in both patients. The sporadic patient carrying the p.G1306E mutation presented at birth with inguinal and hiatal hernia, hypotonia, myotonia with warm-up phenomenon, and laryngeal stridor, which disappeared after 1-year treatment with mexiletine. Permanent weakness was found in the patient with congenital myopathy-like presentation and limited to axial muscles and in the mother with paradoxical myotonia displaying weakness of neck flexor and tongue and facial muscles. Only the three patients with SNEL had cold-induced myotonia, and none of the neonatal *SCN4A* showed painful myotonia.

#### Molecular Genetics

Genetic analysis of *SCN4A* gene in our patient cohort revealed 28 potential disease-causing variants in the 80 patients and two asymptomatic familial cases. These variants involved nine different exons and the most mutated were exons 22, 24, 21, and 13 (Table 2). The most frequent mutations were p.T1313M, p.R1448C/G/H, and p.V1293I found in 18, 13, and 7 patients, respectively (Table 2).

**Table 2:** Panel of the 28 mutations of the SCN4A gene found in our 80 patients.

**Nucleotide change**	**Amino acid change**	**Exon**	**Position on Na_**v**_1.4 channel**	**Phenotype**	**References**	**N. pt**
c.644T>C	p.I215T	5	loop S3DI-S4DI	SCM	Novel	1
c.716T>G	p.I239S	5	loop S4DI-S5DI	SCM	Novel	5
c.825C>A	p.N275K	5	loop S5DI-S6DI	SCM	Novel	1
c.968C>T	p.T323M	6	loop S5DI-S6DI	SCM	SCN4A_000113	1
c.1333G>T	p.V445L	9	loop DI-DII	SCM	Novel	2
c.1333G>A	p.V445M	9	loop DI-DII	SCM	SCN4A_000015	1
c.2006G>A	p.R669H	13	S4DII	HypoPP2	SCN4A_00019	2
c.2023C>G	p.R675G	13	S4DII	Hyper/NormoPP	rs121908556	1
c.2076C>G	p.I692M	13	loop S4DII-S5DII	Hyper/NormoPP	Novel	2
c.2078T>C	p.I693T	13	loop S4DII-S5DII	PMC/Neonatal	SCN4A_00031	3
c.2095G>A	p.A699T	13	S5DII	PMC	rs1057518865	3
c.2111C>T	p.T704M	13	S5DII	Hyper/NormoPP	SCN4A_00033	2
c.3395G>A	p.R1132Q	18	S4DIII	HypoPP2	SCN4A_00043	1
c.3466G>T	p.A1156S	19	loop S4DIII-S5DIII	SCM/asym	Novel	2
c.3491TC	p.L1164P	19	S5DIII	SCM	rs749033669	3
c.3539A>T	p.N1180I	19	loop S5DIII-S6DIII	SCM/Neonatal	(13)	2
c.3877G>A	p.V1293I	21	loop DIII-DIV	SCM	SCN4A_00048	7
c.3890A>G	p.N1297S	21	loop DIII-DIV	SCM	(14)	2
c.3893T>G	p.F1298C	21	loop DIII-DIV	SCM/asym	(14)	2
c.3917G>A	p.G1306E	22	loop DIII-DIV	Neonatal	SCN4A_00050	1
c.3917G>C	p.G1306A	22	loop DIII-DIV	SCM	SCN4A_00051	1
c.3917G>T	p.G1306V	22	loop DIII-DIV	PMC	SCN4A_00052	1
c.3938C>T	p.T1313M	22	loop DIII-DIV	PMC	VAR_001570	18
c.4342C>T	p.R1448C	24	S4DIV	PMC	VAR_001572	6
c.4342C>G	p.R1448G	24	S4DIV	PMC	SCN4A_00070	3
c.4343G>A	p.R1448H	24	S4DIV	PMC	VAR_001573	4
c.4690G>A	p.V1564I	24	loop S5DIV-S6DIV	PMC	rs202106192	2
c.4774A>G	p.M1592V	24	S6DIV	SCM/Hyper/NormoPP	VAR_001575	3

*S, transmembrane segment; D, domain (or repeat); loop, region intra/extra-cellular between 2 segments and 2 domain; SCM, sodium-channel myotonia; PMC, paramyotonia congenita; HypoPP2, hypokalemic periodic paralysis; Hyper/NormoPP, hyperkalemic/normokalemic periodic paralysis; Neonatal, Neonatal SCN4A; asym, asymptomatic; pt, patients*.

*The position of all mutations have been established using NextProt (https://www.nextprot.org/entry/NX_P35499/sequence)*.

Six mutations in the *SCN4*A gene have not been previously reported in the molecular databases (Leiden Open Variation Database, NextProt, The Human Gene Mutation Database, Exome Variant Server and UniProtKB): p.I215T, p.I239S, p.N275K, p.V445L, p.I692M, p.A1156S, p.N1180I, p.N1297S, and p.F1298C (Table 2). Some of these variants were located in the same position or immediately nearby the amino acid of a codon already known to be mutated and associated with disease, or showing pathogenic score, or segregating with disease in familial genetic studies, strongly suggesting that our nine unknown variants are potential disease-causing mutations.

**Figure 1:**
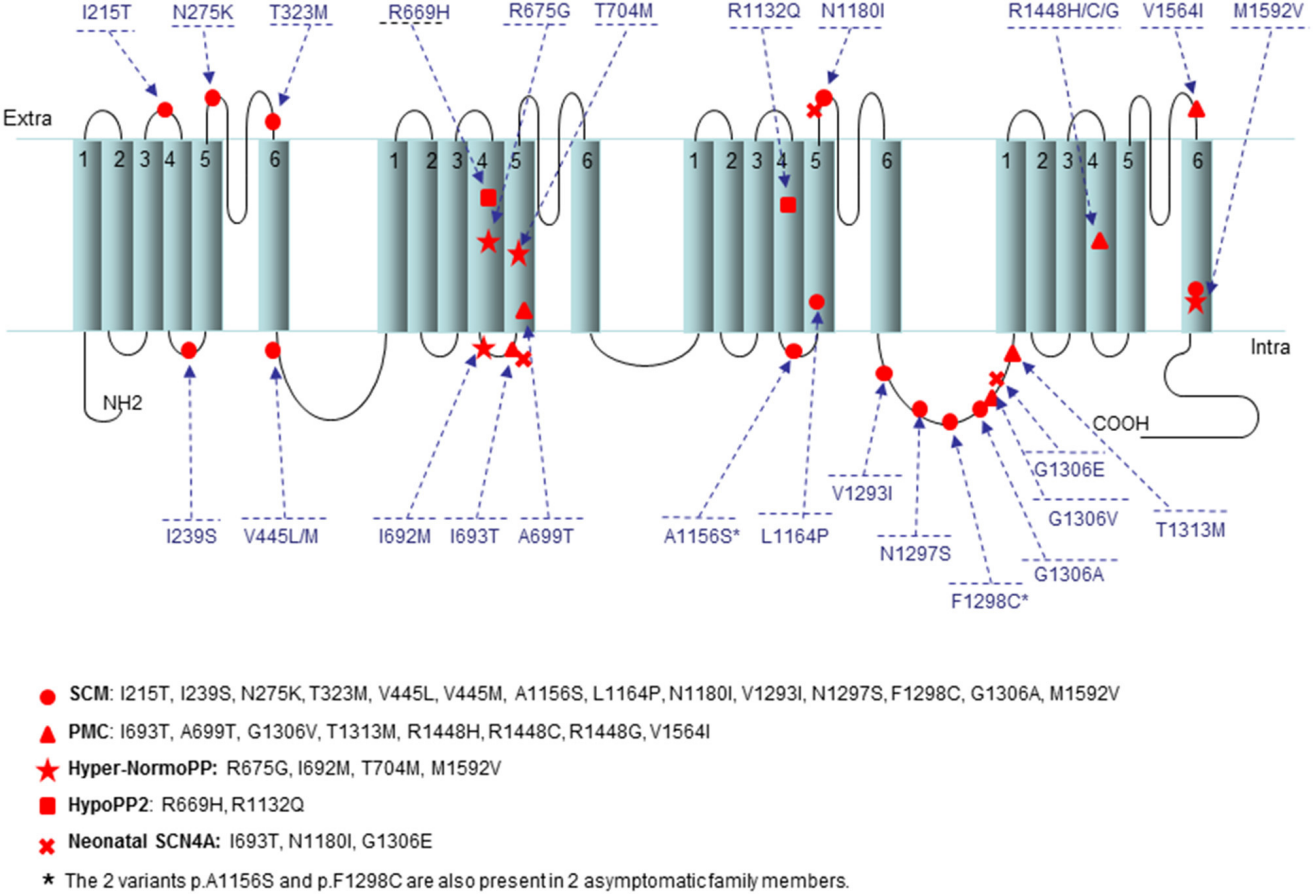
Location of the 28 mutations present in this study onto a secondary structure of Na_v_1.4 channel. The variants are indicated by small circle for SCM, triangle for PMC, star for Hyper/NormoPP, square for HypoPP2, and cross for SNEL. The two variants p.A1156S and p.F1298C are also present in two asymptomatic family members. The position of all variants has been established using NextProt (https://www.nextprot.org/entry/NX_P35499/sequence).

The p.I215T variant present in one SCM patient was placed in the extracellular loop between the transmembrane segments S3 and S4 of the first repeat (loop S3DI-S4DI) (Figure 1). In the same codon the known variant p.I215M (rs373289931) had been previously reported with a pathogenic score (score = 0.98).

The p.I239S mutation detected in 5 SCM patients was located in the extracellular loop between the transmembrane segments S4 and S5 of the first repeat (loop S4DI-S5DI) (Figure 1); in the same loop the variants p.T238K/M and p.V240M (rs201661188 and rs746216167, respectively) had already been reported.

One SCM patient harbored the unreported p.N275K variant localized in the extracellular loop between the transmembrane segments S5 and S6 of the first repeat (loop S5DI-S6DI) (Figure 1); in the previous codon the variant p.G274E had been described (COSM3890178) with a pathogenic score (score 0.99).

The p.V445L mutation found in 2 SCM patients was located in the adjacent N-terminal area of the loop connecting repeats I and II (Figure 1). The novel p.V445L mutation could be pathogenic since the known mutation p.V445M at the same position has been reported in multiple families and individuals affected by myotonia congenita and was associated with marked phenotypic variability. According to the prediction sites the p.V445L also had a pathogenic score (score = 0.82).

In two patients affected by Hyper/NormoPP the p.I692M mutation was placed in the intracellular loop between the transmembrane segments S4 and S5 of the second repeat (loop S4DII-S5DII) (Figure 1), nearby the two known mutations p.I692T and p.I692F (rs757943588 and rs765779586).

The p.A1156S variant found in 1 SCM patient and the asymptomatic father (Table 2) affect the same codon of the p.A1156T mutation (LOVD: SCN4A_00044), which was reported to alter the function of Nav1.4 channel causing a channelopathy, interestingly associated with a mild phenotype without overt myotonia or periodic paralysis but with muscle pain (15).

Three mutations (p.N1180I, p.N1297S, and p.F1298C) found in six patients were already reported and associated to non-dystrophic myotonias (Table 2) (13, 14). Of note, p.N1297S was found in our cohort in two patients also carrying the p.F167L variant in *CLCN1* gene, which probably mitigated the severe effect of the mutation in the Nav1.4 channel, as already reported (8). In our cohort of patients PP was more frequently associated with mutations distributed in the S4 region compared to NDM (*p* < 0.01). No significant association was found between specific domains of Nav1.4 channel and the presence of episodes of paralyses in PMC (*p* = 0.75).

## Discussion

The present study includes a cohort of Italian patients mutated in *SCN4A*, displaying different phenotypes, spanning from SCM and PMC throughHyper/NormoPP and HypoPP2 to Neonatal SCN4A. To our knowledge, this is the first report on a large population of patients investigating clinical features of all these phenotypes together. Skeletal muscle channelopathies associated to *SCN4A* gene mutations indeed represent a continuum in the clinical spectrum, as also supported by the high frequency of episodes of paralysis in PMC and the relative high incidence (50%) of myotonia in Hyper/NormoPP. In addition, features of myotonia and episodes of paralysis did not differ between NDM and PP in terms of muscle distribution and age at onset. Phenotypes were consistent in the same family except for four out of 20 pedigrees, including two asymptomatic patients in late adult life, suggesting relative strong genotype-phenotype correlations in these diseases. On the other hand, clinical heterogeneity could also be a feature of sodium muscle channelopathies, as demonstrated by the p.G1306V/A/E mutations found in three patients displaying three different phenotypes (PMC, SCM, and SNEL), demonstrating how the replacement of glycine at the same codon of the Na_V_1.4 with three different amino acids (Valine/Alanine/Glutamic Acid) may lead to different phenotypes. Hence, other mechanisms, such as the coexistence of *SCN4A* and *CLCN1* gene mutations and non-genetic factors (epigenetic, environmental, and hormonal), should be considered to explain the clinical variability in these disorders (8, 16).

In our cohort PMC was the most frequent phenotype, accounting for almost a half of the whole population, followed by SCM and then by PP and Neonatal *SCN4A*, which represent a minority of the cases. Our data showing greater frequency of NDM than PP caused by *SCN4A* gene mutations are in agreement with previous studies on large cohorts of British and Dutch patients (5, 17), although in our cohort PP seems to be underrepresented, being only 12.5% of the population.

In our study, PMC appeared to be significantly associated to earlier onset and cold-sensitivity than SCM and tended to affect more frequently cranial and hand muscles; conversely, SCM tended to show more frequently lower limb and painful myotonia and muscle hypertrophy than PMC, in agreement with previous reports (2, 6, 7). Hyper/NormoPP had earlier onset than HypoPP2 and tended to be associated more frequently with focal and shorter episodes of paralysis; myotonia and muscle hypertrophy were found only in Hyper/NormoPP patients (6, 18, 19). On the other hand, HypoPP2 displayed more frequently generalized PP than Hyper/NormoPP. Permanent weakness still represents an issue in skeletal muscle channelopathies, mainly due to the poor and somehow conflicting data on its frequency, presentation, and progression over time; in addition, its pathomechanisms are still not completely elucidated (20, 21). In our cohort, permanent weakness was detected in about one quarter of the patients, without any significant difference between NDM and PP or SCM and PMC. Of note, permanent muscle weakness was observed with neurological examination in about half of Hyper/NormoPP patients and in none of HypoPP2 patients, as previously reported (6). Muscle weakness was limited to thigh muscle in Hyper/NormoPP and more diffuse in NDM, including also cranial, axial, and distal upper limb muscles, with the latter appearing to be specific for PMC. To date, no predictive factors for permanent weakness are known and this phenomenon seems to occur regardless of the mutation.

Neonatal-*SCN4A* represents 5% of the whole cohort. There is a partial overlap with congenital myopathies due to the presentation at birth with hypotonia in three out four patients and bilateral clubfoot, hip dislocation, facial dysmorphism, or inguinal and hiatal hernia in two out of four patients. Autosomal recessive mutations in *SCN4A* gene have been reported in association with severe congenital myopathy without myotonia (22), while patients with simple heterozygous mutations may represent a milder phenotype associated with myotonia presenting at birth or in 1st years of age (23–26). Notably, myotonia in neonatal SCN4A may be associated to warm-up phenomenon or paradoxical, depending on the causing mutations, mainly the p.G1306E and p.I693T, respectively (23–25). Although the SNEL cases reported in literature are usually sporadic, we report here two familial cases (mother and son) with autosomal dominant inheritance displaying this phenotype.

The alpha-subunit of the voltage-gated sodium channel Na_V_1.4 is composed of four highly homologous domains (DI-DIV) each consisting of six transmembrane segments (S1–S6). When inserted in the membrane, the four repeats form a central pore with segments five and six lining its wall (Figure 1). The repeats are connected by intracellular loops; one of them is in the III–IV linker which contains the fast-inactivation particle and many mutations found in our population are located in this region, such as the frequent p.T1313M mutation found in 18 patients (Table 2). Notably, we found seven missense mutations (p.V1293I, p.N1297S, p.F1298C, p.G1306V/A/E, and p.T1313M) in this relevant portion of the Na_V_1.4 in 32 patients affected by different myotonic phenotype (SCM, PMC, and SNEL) and with various degrees of severity (Table 2, Figure 1). A further important region for the functionality of the Na_v_1.4 channel is the voltage sensor localized in the transmembrane segment S4. In this region we found six mutations: p.R669H and p.R675G in the second domain, p.R1132Q in the third domain, and p.R1448C/G/H in the fourth repeat (Figure 1).

Both the p.R669H and p.R1132Q mutations found in 3 HypoPP2 patients cause a change of an arginine in S4 segments of the second and third repeats, respectively; conversely, as also reported by Cannon (1). In addition, the p.R675G variant found in 1 patient affected by Hyper/NormoPP, and the mutations p.R1448C/G/H in 13 patients affected by PMC, further confirm that the S4 is an essential region for the function of the sodium channel and that changes in this transmembrane segment are associated with a large phenotypic variability. These mutations may increase the continuous current of the sodium channel, change the voltage-dependent activation process, lead to abnormal depolarization of resting potential, or slow down the inactivation process, affecting the normal function of sodium channel and leading to disease. However, mutations in the S4 segments appeared to be significantly more frequent in PP than NDM patients.

Based on the distribution of mutations along the *SCN4A* gene, we may conclude that the hot-spot regions for sodium channelopathies in our cohort patients involve the exons 13, 21, 22, 24, in agreement with the literature (27, 28). These exons encoding the S4–S5 segments of the second domain, the loop between the third and the fourth domain and the segments S4 and S6 of the fourth domain, as shown in Figure 1 and Table 2.

Genetic analysis of *SCN4A* gene in our cohort patients revealed a greater prevalence of PMC-associated mutations in the C-terminal region of the Na_V_1.4 channel protein than the more diffuse distribution of SCM-associated mutations, detected in all 4 domains of the protein (Table 2, Figure 1).

In conclusion, our data provide further insight in the field of skeletal muscle channelopathies due to mutations in *SCN4A*; however, prospective studies on large cohort of patients are needed to better clarify the natural history of these diseases and investigate possible genetic and non-genetic modifiers of the phenotype.

## Data Availability Statement

The datasets generated for this study can be found in the https://databases.lovd.nl/shared/individuals/SCN4A.

## Ethics Statement

The studies involving human participants were reviewed and approved by Ethics Committee at Fondazione IRCCS Istituto Neurologico Carlo Besta. Written informed consent to participate in this study was provided by the participants' legal guardian/next of kin.

## Author Contributions

LM, RB, and PB conceived and designed the study. LM, RB, PB, RM, and IT drafted manuscript, figure, and tables. All the authors acquired and analyzed the data and gave the final approval to the current version of the manuscript.

## Conflict of Interest

The authors declare that the research was conducted in the absence of any commercial or financial relationships that could be construed as a potential conflict of interest.
